# A Novel Multiplexed, Image-Based Approach to Detect Phenotypes That Underlie Chromosome Instability in Human Cells

**DOI:** 10.1371/journal.pone.0123200

**Published:** 2015-04-20

**Authors:** Laura L. Thompson, Kirk J. McManus

**Affiliations:** 1 Department of Biochemistry and Medical Genetics, University of Manitoba, Winnipeg, Manitoba, Canada; 2 Manitoba Institute of Cell Biology, Winnipeg, Manitoba, Canada; University of Science and Technology of China, CHINA

## Abstract

Chromosome instability (CIN) is characterized by a progressive change in chromosome numbers. It is a characteristic common to virtually all tumor types, and is commonly observed in highly aggressive and drug resistant tumors. Despite this information, the majority of human CIN genes have yet to be elucidated. In this study, we developed and validated a multiplexed, image-based screen capable of detecting three different phenotypes associated with CIN. Large-scale chromosome content changes were detected by quantifying changes in nuclear volumes following RNAi-based gene silencing. Using a DsRED-LacI reporter system to fluorescently label chromosome 11 within a human fibrosarcoma cell line, we were able to detect deviations from the expected number of two foci per nucleus (one focus/labelled chromosome) that occurred following CIN gene silencing. Finally, micronucleus enumeration was performed, as an increase in micronucleus formation is a classic hallmark of CIN. To validate the ability of each assay to detect phenotypes that underlie CIN, we silenced the established CIN gene, *SMC1A*. Following *SMC1A* silencing we detected an increase in nuclear volumes, a decrease in the number of nuclei harboring two DsRED-LacI foci, and an increase in micronucleus formation relative to controls (untreated and si*GAPDH*). Similar results were obtained in an unrelated human fibroblast cell line. The results of this study indicate that each assay is capable of detecting CIN-associated phenotypes, and can be utilized in future experiments to uncover novel human CIN genes, which will provide novel insight into the pathogenesis of cancer.

## Introduction

Cancer is a significant global concern with more than 14 million new diagnoses and over 8 million deaths attributed to this disease each year[1]. In order to develop superior therapeutic strategies to improve cancer treatments, it is essential that we gain a greater understanding of the etiologic origins and aberrant molecular mechanisms that drive tumorigenesis. Chromosome instability (CIN) is a hallmark of cancer that occurs frequently in both solid (e.g. colorectal, breast, prostate) and liquid (lymphoma and leukemia) tumors[2–7]. CIN is characterized by an increase in the rate at which whole chromosomes or large chromosomal fragments are gained or lost, and typically manifests as abnormal chromosome numbers or aneuploidy[2, 8, 9]. It is predicted to occur early in cancer development, and act as a driving force in tumor progression by increasing the rate at which oncogenes or tumor suppressor genes are gained or lost, respectively[7, 8]. In addition, CIN is associated with highly aggressive tumors[10], the acquisition of multi-drug resistance[11, 12], tumor recurrence[13], and consequently poor patient prognosis[14]. Despite these associations, the aberrant molecular origins (i.e. aberrant genes) that cause CIN remain largely unknown[7]. Therefore, identifying the altered or misregulated genes that underlie CIN is critical to gain a greater understanding of their potential role(s) in the tumorigenic process.

As chromosome stability is essential for the survival of all living organisms, many of the genes and biological processes required to maintain chromosome stability are inherently conserved across species. In model organisms such as *Saccharomyces cerevisiae*, CIN genes have been identified through the use of complementary assays capable of detecting increases in one or more CIN-associated phenotypes including whole chromosome loss, gene conversion, and/or chromosomal rearrangements[15, 16]. Using these approaches, Stirling *et al*[16] recently identified a total of 692 genes necessary for the maintenance of chromosome stability, which represents ~11.5% of the gene complement from *S*. *cerevisiae* (~6,000 total genes). If a similar frequency is observed in humans (~20,000 total genes), more than ~2,300 CIN genes are predicted to exist, however only a small fraction have been identified to date[9, 17–19]. Accordingly, identifying and developing novel approaches to screen large numbers of candidate genes are highly warranted, as they will ultimately shed novel insight into the genes and mechanism(s) normally required to ensure chromosome stability in humans.

The underlying aberrant phenotypes that drive CIN are complex and heterogeneous. They can be caused by the misregulation of many biological processes including sister chromatid cohesion, centrosome biology, cell cycle checkpoints, and DNA damage repair (reviewed in [8, 20]). Sister chromatid cohesion for example, is established following DNA replication (reviewed in [21]), and is mediated by the cohesin complex and accessory proteins. Its main function is to prevent premature chromatid separation, and thus cohesion is normally required to ensure proper chromosome segregation and stability during mitosis[22]. Studies have shown that diminished expression of cohesion-related genes including the cohesin subunit, *SMC1A*, are associated with chromosome content changes that are characterized by an increase in the number of tri- and tetraploid cells [5, 18, 23, 24]. More recently, SMC1A and the cohesin complex have demonstrated additional roles in centrosome dynamics by invoking a DNA damage-induced cell cycle checkpoint, and in the DNA damage repair process itself[25–27]. Defects in these pathways may result in global chromosome content changes, but may also manifest as smaller-scale changes involving individual chromosomes, or chromosomal fragments following DNA damage. Although small-scale chromosome content changes may not have a significant impact on overall nuclear volume, lagging chromosomes or acentric chromosomal fragments that fail to incorporate into one of the daughter nuclei following division, may form micronuclei[28]. Micronuclei are considered a hallmark of CIN and are frequently observed in cancer[29–31]. Thus, the presence of micronuclei, or their induced formation can be used as a surrogate marker for CIN.

Despite what is known about the mechanisms underlying CIN, relatively few human CIN genes have been identified. The gaps in our knowledge are attributed at least in part, to the lack of highly efficient methodologies capable of detecting CIN. Traditional cytogenetic approaches, including chromosome enumeration within mitotic chromosome spreads, are laborious, time consuming, costly, and unsuitable for the high-throughput screening of hundreds to thousands of candidate genes[18, 32, 33]. These limiting aspects highlight the need for novel CIN detection methods that are amenable to rapid, high-content screening, to identify maximal numbers of novel CIN genes in humans. A recent body of evidence has begun to emerge which suggests nuclear volume may be an excellent surrogate marker for CIN. Conceptually, large increases in chromosome numbers (i.e. ploidy) will be reflected by corresponding increases in nuclear volume. Indeed, studies evaluating the relationship between DNA content and nuclear size have generally revealed a positive correlation[34–37]. However, CIN is not simply defined by increases in ploidy, as it also includes more subtle increases involving one or a few chromosomes. Presumably, smaller changes in chromosome content can be detected through the incorporation of a chromosome-specific marker such as a *Lac Operator* (*LacO*) cassette[38–40], which is visualized by a fluorescently tagged lac repressor protein (LacI), and gains or losses in copy number are indicative of CIN. Alternatively, mis-segregated whole chromosomes or acentric chromosome fragments are expected to form micronuclei that are easily detected using standard DNA counterstains such as Hoechst[29, 41]. Thus, a screen capable of rapidly and simultaneously assessing these phenotypes would dramatically increase the speed at which CIN genes are identified.

In this study, we develop and validate a multiplexed and image-based approach capable of detecting three phenotypes associated with CIN. The nuclear volume assay monitors changes in nuclear size as an indicator of large-scale chromosome content changes associated with CIN. The foci enumeration assay utilizes a DsRED-LacI reporter system to monitor a *LacO* cassette integrated within chromosome 11[38], and assesses small-scale copy number changes involving a single chromosome. Finally, the micronucleus (MN) enumeration assay detects the loss of whole chromosomes or large chromosomal fragments derived from DNA double-strand breaks and/or segregation defects. Each assay was validated through the use of established positive (SMC1A) and negative (GAPDH) controls[18, 21]. Following *SMC1A* silencing, statistically significant increases in mean nuclear volume were readily detected. Decreases in the number of nuclei harbouring the expected two DsRED-LacI foci, and increases in MN formation were also successfully detected. When employed in a different cellular context, similar results were obtained. These data validate the use of this multiplexed screening approach to identify phenotypes associated with CIN and thus CIN genes themselves.

## Materials and Methods

### Cell Lines and Culture

J21 cells were generously provided by Dr. J. Chubb (University College, London), and are a karyotypically stable, human HT1080 fibrosarcoma cell line containing 20–30 copies of a *LacO* cassette (~128-mer) integrated at 11q13[38–40]. Cells and the presence of the *LacO* cassettes were validated through mitotic spreads and karyotypic analyses (see below). The J21 subclone was confirmed to harbor two copies of the *LacO* cassette, one copy per chromosome 11. J21 cells were cultured in DMEM/High Glucose Media (HyClone) containing blasticidin (2.5 μg/mL), puromycin (0.5 μg/mL) and 10% fetal bovine serum (FBS). The karyotypically stable[42], immortalized (telomerase), human fibroblast cell line hTERT[43], was generously provided by Dr. C. P. Case (University of Bristol) and grown in DMEM (HyClone) media supplemented with 10% FBS. Cell lines were authenticated on the basis of recovery, viability, growth, morphology and spectral karyotyping as detailed elsewhere[42]. All cells were grown in a 37°C humidified incubator with 5% CO_2_.

### Generation of DsRED-LacI J21 Cells

A LacI expression plasmid was provided by Dr. J. Chubb[38]. *LacI* was PCR amplified, and sub-cloned into the pLVX-DsRED-Monomer-C1 (Clontech) using the InFusion HD (Clontech) system as instructed by the manufacturer. The vector was sequence verified (McGill University and Genome Quebec). Cells were virally transduced and DsRED-LacI expressing clones were selected using a standard drug selection protocol (0.5 μg/mL puromycin).

### Confirmation of the *LacO* Integration Loci

Asynchronous J21 cells were harvested, fixed, treated with KCl (hypotonic treatment) and cytospun on to glass slides. Mild-treatment conditions were utilized to preserve protein interactions between the DsRED-LacI and the *LacO* cassettes. Chromosomes were DAPI-counterstained, and mitotic chromosome spreads were subjected to spectral imaging using an Applied Spectral Imager (ASI) to ensure DAPI and DsRED-LacI signal intensities were clearly separated. The position and number of DsRED-LacI foci were noted for a minimum of 40 mitotic chromosome spreads.

### Evaluating the Karyotypic Stability of the *LacO* Cassettes

Cellular aliquots were harvested from a long-term growing and untreated culture of J21 cells at two timepoints (t = 0 and 6 weeks). Briefly, each aliquot was dispensed onto glass coverslips and the cells were permitted to attach. Cells were fixed, counterstained with Hoechst, imaged, and the total number of interphase nuclei harboring two DsRED-LacI foci was determined from a minimum of 100 cells.

### Gene Silencing

Cells were transiently transfected with siRNA duplexes using RNAiMax (Invitrogen) as described elsewhere[18]. ON-TARGETplus siRNA duplexes targeting *SMC1A* and *GAPDH* were purchased (Dharmacon) and employed as either individual siRNAs (100 nM total) or as a pool comprised of four unique siRNAs (25 nM each or 100 nM total) targeting distinct regions of the coding sequence. Gene silencing was confirmed by standard Western blots (see below) four days post-transfection.

### Western Blot Analysis

Proteins were harvested from each treatment condition as described previously[18]. Membranes were blotted with rabbit anti-SMC1A primary antibody (Abcam; ab9262; [1:10,000]). Blots were stripped and re-blotted with mouse monoclonal anti-α-tubulin antibody (Abcam; ab7291; [1:4000]) as a loading control. All primary antibodies were visualized by secondary antibodies conjugated to horseradish peroxidase. Blots were imaged and bands were visualized on a MyECL Imager (Thermo Scientific) using standard chemiluminescence.

### Fluorescence Microscopy

Cells were transfected as described above (*Gene Silencing*), fixed 4-days post-transfection with 4% paraformaldehyde, counterstained with DAPI, and 3D images were collected as detailed elsewhere[5, 44]. Briefly, images were acquired using an AxioImager Z1 Microscope (Zeiss) equipped with an AxioCam HR charge-coupled device (CCD) camera (Zeiss), and a 20X dry plan-neofluar objective (0.5 numerical aperture). The exposure times were first optimized for each channel, and then set and maintained constant throughout the entire image acquisition phase. Approximately 25 optical sections were acquired at 0.400 μm intervals using DAPI and Cy3 filters to acquire nuclear and DsRED-LacI data, respectively. 3D images were imported into AutoQuant X3 (Media Cybernetics) and subjected to maximum-likelihood-expectation deconvolution using a constrained iterative algorithm and theoretical point spread functions for the DAPI (461 nm) and Cy3 (570 nm) channels. Each 16-bit image was imported into Imaris v7.7.1 (Bitplane) image visualization software and analyzed as detailed below. Representative images were exported into Photoshop CS6 (Adobe) where figure panels were assembled.

### Nuclear Volume Assay

Imaris software was employed to automatically generate 3D surface renderings of interphase nuclei (based on the DAPI signal intensity) from which corresponding nuclear volumes were determined and compared between conditions (untreated, si*GAPDH* and *siSMC1A*). To ensure only complete nuclei were included in the analyses, an XY boundary exclusion filter (<8 μm) was employed to remove partially intact nuclei located along the image periphery, while volume (>500 μm^3^) and mean DAPI intensity (<1.0×10^4^ au) inclusion filters were employed to eliminate small nuclear debris (i.e. apoptotic bodies) and mitotic cells, respectively. Nuclear volume data from a minimum of 200 nuclei were generated for each condition and exported into Prism v6 (GraphPad), where standard statistical analyses (e.g. mean, standard deviation) were performed, and graphs (e.g. box-and-whisker, column, and dot plots) were generated. Student’s *t*-tests were employed to compare the mean nuclear volumes of the controls (untreated and si*GAPDH*) and the experimental condition (si*SMC1A*), and a *p*-value of <0.05 was deemed statistically significant.

### DsRED-LacI Foci Enumeration Assay

The number of DsRED-LacI foci was manually enumerated from a minimum of 200 nuclei per condition. The number of nuclei exhibiting one, two, and more than two nuclear foci were calculated and expressed as a percentage of the total number of nuclei analyzed for each condition. In addition, a CIN score (CS) was calculated, which is a metric used to describe both the gains and losses of DsRED-LacI foci from the expected number of two per nucleus. For a given nucleus, the CS is calculated according to [CS = |*e - o*|], where CS equals the absolute value obtained when the observed number of DsRED-LacI foci (*o*) is subtracted from the expected number of two DsRED-LacI foci (*e*). To calculate the mean CS ($\overset{-}{CS}$) for a given population, the absolute values within a specific condition are summed and divided by the total number of nuclei evaluated according to $\left[\bar{CS}=\frac{1}{n}\overset{n}{\underset{i=1}{\sum }}\left({\left|e-o\right|}_{i}\right)\right]$. Mann-Whitney tests were performed in Prism to compare the CS distributions calculated for si*GAPDH* and si*SMC1A* cells with untreated controls. Graphs were generated in Prism, and figure panels were assembled in Photoshop.

### Micronucleus Enumeration Assay

3D images were imported into Imaris software where micronuclei were automatically scored for each condition. Micronuclei were defined as small (<1/3 the size of the nucleus), extra-nuclear DAPI-stained bodies exhibiting no visible attachments with the primary nucleus[29]. Micronuclei were distinguished from bright DAPI-stained apoptotic bodies, which were eliminated from analysis using an intensity threshold exclusion filter (mean signal intensity >1.0x10^4^ a.u.). For each condition the number of micronuclei was expressed as a percentage of the total number of nuclei analyzed. MN data were imported into Prism where statistical analyses were performed and graphs were generated as above.

### Mitotic Spreads and Chromosome Enumeration

Mitotic chromosome spreads were generated from hTERT cells for each condition (untreated, si*GAPDH*, and si*SMC1A*) as detailed elsewhere[5, 18]. Briefly, subconfluent cells were mitotically-enriched using KaryoMAX colcemid (0.1 mg/ml; Gibco) for 4 h prior to harvesting. Cells were treated for 8 min in hypotonic solution (75mM KCl) and fixed using three 10 min washes with methanol:acetic acid (3:1). Chromosomes were counterstained with DAPI, and 100 spreads per condition (untreated, siGAPDH and siSMC1a) were imaged using an AxioImager Z1 microscope equipped with a 63× oil immersion plan apochromat lens (1.4 numerical aperture) and a Zeiss HRm CCD camera. 16-bit TIF images were acquired and imported into ImageJ software where chromosomes were manually enumerated. To evaluate statistical differences in the distribution of chromosome contents between conditions, two-sample Kolmogorov-Smirnov tests were performed (Prism).

## Results

### Increases in Nuclear Volumes are Associated with Silencing of a CIN Gene

**Fig 1:**
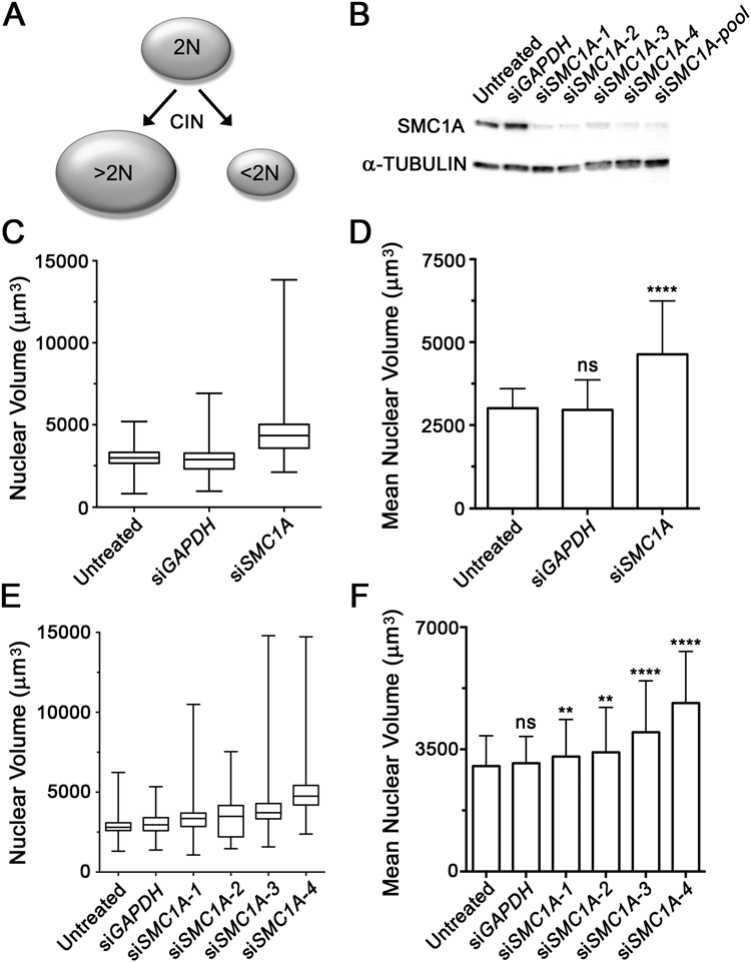
*SMC1A* Silencing Underlies Increases in Nuclear Volumes in J21 Cells. (A) A conceptual schematic depicting the relationship between changes in nuclear volume (ovals) and DNA content (2N) that are predicted to occur due to chromosome mis-segregation events occurring during cellular division. (B) Western blot depicting *SMC1A* silencing following treatment with individual (si*SMC1A-1*, -*2*, -*3* and -*4*) or pooled (si*SMC1A-pool*) siRNA duplexes relative to controls (untreated and si*GAPDH*). α-TUBULIN serves as the loading control. (C) Box-and-whisker plot depicting the distribution range of nuclear volumes measured for the indicated conditions (x-axis). Whiskers delineate the entire distribution range, while the lower, middle and upper horizontal lines of the box identify the 25^th^, 50^th^ and 75^th^ percentiles, respectively. (D) Bar graph presenting the mean nuclear volumes ± standard deviation (SD) measured for the indicated conditions (x-axis). Highly statistically significant increases in mean nuclear volumes were observed following *SMC1A* silencing (*p*-value <0.0001; ****) relative to the untreated controls that were not significant (*p*-value >0.05; ns) following *GAPDH* silencing. (E) Box-and-whisker plot depicting the total distribution range and 25^th^, 50^th^ and 75^th^ percentiles of nuclear volumes measured for each of the individual siRNA duplexes targeting *SMC1A* or controls. (F) Bar graph depicting the mean nuclear volume ± SD following silencing. Student’s *t*-tests between untreated controls and each condition revealed statistically significant increases (*p*-value <0.01; **: *p*-value <0.0001; ****) in mean nuclear volumes following *SMC1A* silencing that were not significant (*p*-value >0.05) following *GAPDH* silencing (si*GAPDH*).

Previous studies utilizing human tumor samples[34–37] have shown a positive correlation between increases in chromosome number and nuclear size. However, this concept has never been applied in the context of a screen in cell lines. To determine whether changes in nuclear volume may act as a surrogate marker of CIN (Fig 1A), we first sought to silence an established CIN gene (*SMC1A*) that can induce large increases in chromosome complements (i.e. ploidy)[18]. However, prior to performing the volumetric analyses, the silencing efficiencies of the four individual siRNA (si*SMC1A*-1, -2, -3 and -4) and pooled (*SMC1A*-pool) duplexes were evaluated (Fig 1B). Having established that all siRNA conditions efficiently silence SMC1A, karyotypically stable J21 cells were transiently transfected using a pooled siRNA approach. Four days post-transfection, cells were fixed, counterstained with DAPI and subjected to 3D image acquisition and analysis as detailed within Materials and Methods. Briefly, the DAPI channel (which fluorescently labeled nuclei) was employed to generate surface renderings for each nucleus within each deconvolved image. Nuclear volumes were automatically determined for a minimum of 200 nuclei per condition, and statistical comparisons were made.

As predicted, decreased expression of SMC1A was accompanied by qualitative and quantitative changes in nuclear volume in J21 cells. More specifically, an increase in the total distribution range of the nuclear volumes (i.e. minimum to maximum) was observed within the *SMC1A*-silenced cells (total range = 11,735 μm^3^) relative to untreated (4,397 μm^3^) or *GAPDH*-silenced (5,967 μm^3^) controls (Fig 1C). In addition, a 1.5-fold increase in mean nuclear volume occurred within the *SMC1A*-silenced cells (4,632.6 ± 1,608.7 μm^3^ [SD]) relative to untreated (3,008.1 ± 592.6 μm^3^) or *GAPDH*-silenced (2,955.9 ± 907.7 μm^3^) controls (Fig 1D). Student’s *t*-tests revealed this increase to be highly statistically significant compared to the untreated (*p*-value <0.0001) (Fig 1D) or *GAPDH*-silenced (*p*-value <0.0001) cells (S1 Table).

To address potential off-target effects associated with the pooled siRNA approach, each individual duplex was also evaluated for its ability to induce changes in nuclear volume. Having established each individual siRNA duplex efficiently silences SMC1A (Fig 1B), we now employed a similar experimental approach to that used above. In agreement with the pooled approach, silencing of SMC1A expression by each of the individual siRNA duplexes was associated with increases in the total distribution range of nuclear volumes (Fig 1E), increases in nuclear volumes, and statistically significant increases in the mean nuclear volumes relative to controls (Fig 1F and S2 Table). Although differences were observed between each of the individual SMC1A duplexes, this was expected and is likely attributed to the differences in silencing efficiency of each duplex, or more likely, the heterogeneous nature of the CIN phenotype itself. Collectively, the above data show that *SMC1A* silencing is accompanied by increases in nuclear volumes and therefore supports the use of nuclear volumes as surrogate markers for CIN in J21 cells.

To confirm the alterations in nuclear volumes observed above were not restricted to J21 cells, similar experiments were performed in hTERT, an immortalized and karyotypically stable, human fibroblast cell line. As above, Western blots confirmed the silencing of SMC1A following transient transfection with siRNA duplexes (S1 Fig). In agreement with the J21 data, an overall increase in the total distribution range of the nuclear volumes was observed following *SMC1A* silencing (total range = 6,546 μm^3^), relative to the untreated (4,301 μm^3^) or *GAPDH*-silenced (5,096 μm^3^) controls (S1 Fig). *SMC1A* silencing was also associated with a highly statistically significant, 1.3-fold increase in mean nuclear volume (3,539.5 ± 1225.0 μm^3^) relative to untreated (2,640.7 ± 740.8 μm^3^; *p*-value <0.0001) and *GAPDH*-silenced (2,720.1 ± 959.1 μm^3^; *p*-value <0.0001) controls (S1 Fig and S3 Table). These data indicate that the changes in nuclear volume following *SMC1A* silencing are conserved within an hTERT cellular context. The underlying chromosome content changes stemming from *SMC1A* silencing were validated from mitotic chromosome spreads generated in hTERT cells. Following *SMC1A* silencing, 42% of mitotic spreads exhibited an abnormal chromosome number (≠ 46), which represents a 3.8- to 4.7-fold increase relative to untreated (11%) or si*GAPDH*-treated (9%) hTERT cells, respectively, which was determined to be statistically significant (S4 Table). Collectively, the above data show that silencing *SMC1A* induces statistically significant increases in nuclear volumes and validates the ability of the nuclear volume assay to detect changes that are indicative of CIN.

### *SMC1A* Silencing Alters the Number of DsRED-LacI Foci in J21 Cells

Having established an image-based assay capable of quantifying large-scale changes in DNA, we now wished to develop an approach capable of detecting alterations involving a single chromosome. To accomplish this, we obtained J21 cells harboring an *Escherichia coli LacO* cassette (20 to 30 copies) at 11q13[38], which is visualized through the binding of a fluorescently tagged Lac repressor protein (DsRED-LacI). However, prior to employing the J21 cells, we first confirmed the position and number of *LacO* integration sites within mitotic chromosome spreads. Visual inspection of 40 spreads confirmed that the DsRED-LacI foci were uniquely associated with chromosome 11, were associated with both copies of chromosome 11 (i.e. two copies/spread), and localized to a pericentric region along the long (q) arm of chromosome 11 (i.e. 11q13) (S2 Fig). Next, we confirmed the karyotypic stability of chromosome 11 from a continuously growing and untreated population of J21 cells at two distinct timepoints (t = 0 and 6 weeks). As shown in S5 Table, the number of interphase cells harboring two DsRED-LacI foci was consistent and ~75–80% at both timepoints indicating that the number of DsRED-LacI foci, and consequently the copy number of chromosome 11, was stable for at least six weeks.

**Fig 2:**
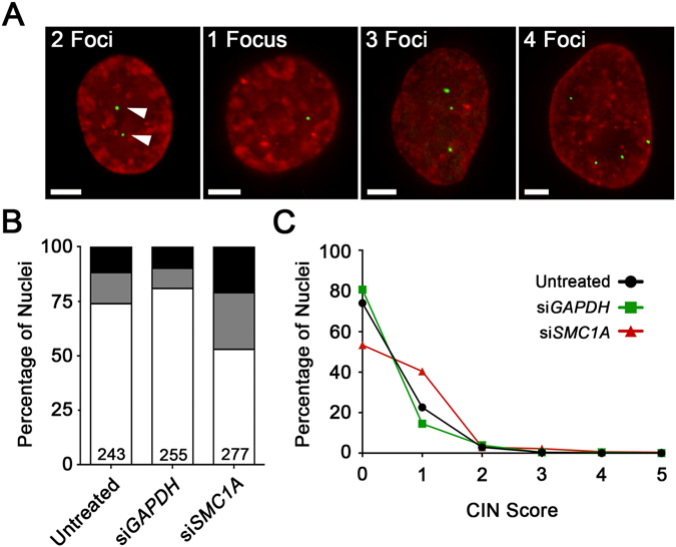
*SMC1A* Silencing Alters Chromosome 11 Copy Number. (A) Representative examples of high-resolution, 3D image projections of DsRED-LacI foci (green) within interphase nuclei (red) from J21 cells. The expected number of two DsRED-LacI foci (left; arrowheads) are frequently either lost (e.g. one focus/nucleus) or gained (e.g. three or four foci/nucleus) following *SMC1A* silencing. Scale bar represents 5 μm. (B) Bar graph presenting the percentage of nuclei harboring the expected number of two foci/nucleus (white), relative to those with losses (gray) or gains (black) in foci. The number of nuclei evaluated is indicated at the base of the column. (C) Histogram presenting the distribution of CIN scores in untreated (black circles), *GAPDH* (green squares) and *SMC1A* (red triangles) silenced cells. Note that a CIN score = 0 indicates that a cell harbors the expected number of two DsRED-LacI foci.

Having confirmed the presence and karyotypic stability of the *LacO* cassette, we now sought to determine whether changes in chromosome 11 copy numbers (i.e. DsRED-LacI foci) could be used as a surrogate marker for CIN following *SMC1A* silencing. Conceptually, increases or decreases in DsRED-LacI foci from the expected number of two foci per nucleus (i.e. one per chromosome) reflect gains or losses of chromosome 11, respectively (Fig 2A). Having already confirmed our ability to silence *SMC1A* with siRNAs (Fig 1B), J21 cells were transiently transfected using the pooled approach described above. Four days post-transfection, cells were fixed, counterstained with DAPI, imaged, and the number of DsRED-LacI foci was evaluated from a minimum of 200 nuclei for each condition. In general, *SMC1A* silencing was associated with alterations in the expected number of DsRED-LacI foci. While 74.1% (untreated) and 80.8% (*GAPDH*-silenced) of the negative control cells harboured the expected two DsRED-LacI foci respectively, only 53.4% of *SMC1A*-silenced cells exhibited two foci per nucleus (Fig 2B), representing a 1.4 to 1.5-fold decrease. In addition, there was a corresponding increase in the overall percentage of *SMC1A*-silenced cells that lost (25.6%) or gained (21.0%) DsRED-LacI foci, compared with untreated (13.6% and 12.3%, respectively) and *GAPDH*-silenced (9.0% and 9.8%) cells (Fig 2B). These data show that gains and losses in DsRED-LacI foci from the expected two per nucleus occur more frequently in *SMC1A*-silenced cells compared to controls.

As the predominant proportion of cells with aberrant focal counts typically harbored single gains or losses in DsRED-LacI foci, simply calculating a mean focal number would not reveal statistically meaningful data. To better quantify the gains and losses of chromosome 11q13 (i.e. DsRED-LacI foci), a CS was developed (see Materials and Methods). By definition, cells with a CS = 0 are diploid for chromosome 11 while those with a CS > 0 exhibit copy number variation with larger CS values and thus larger $\overset{-}{CS}$ values, reflecting a greater degree of chromosome 11 gain or loss. In agreement with the nuclear volume data, an increase in $\overset{-}{CS}$ was observed within *SMC1A*-silenced cells that is ~2-fold greater than controls (Table 1). There was also a large decrease in the proportion of cells with two copies of chromosome 11 (CS = 0), and a corresponding increase in those with either a single gain or loss in chromosome 11 (CS = 1) within the *SMC1A*-silenced cells relative to controls (Fig 2C). Moreover, a modest increase in the percentage of cells with CS ≥ 2 was observed within the *SMC1A*-silenced cells (6.1%) relative to untreated (3.3%) or *GAPDH*-silenced (4.7%) controls. The Mann-Whitney test confirmed statistically significant differences in the distribution of CS values in the SMC1A silenced cells compared to the negative controls (Table 1). Collectively, these data show that the DsRED-LacI foci enumeration assay can be employed as an effective indicator of CIN in J21 cells.

**Table 1:** Comparison of CIN Scores to Untreated Cells.

	# Nuclei	Mean CIN Score (a.u.)	Standard Deviation	Fold Increase^A^	*p*-value^B^
**Untreated**	243	0.296	0.540	**N/A**	**N/A**
**si*GAPDH***	255	0.251	0.582	0.85	0.1045
**si*SMC1A***	277	0.574	0.770	1.94	<0.0001

^A^Fold increase values refer to the increase in mean CS relative to untreated control.

^B^Mann-Whitney tests comparing the distribution of CS values between conditions. A *p*-value <0.05 is considered statistically significant.

### *SMC1A* Silencing Induces Micronucleus Formation

**Fig 3:**
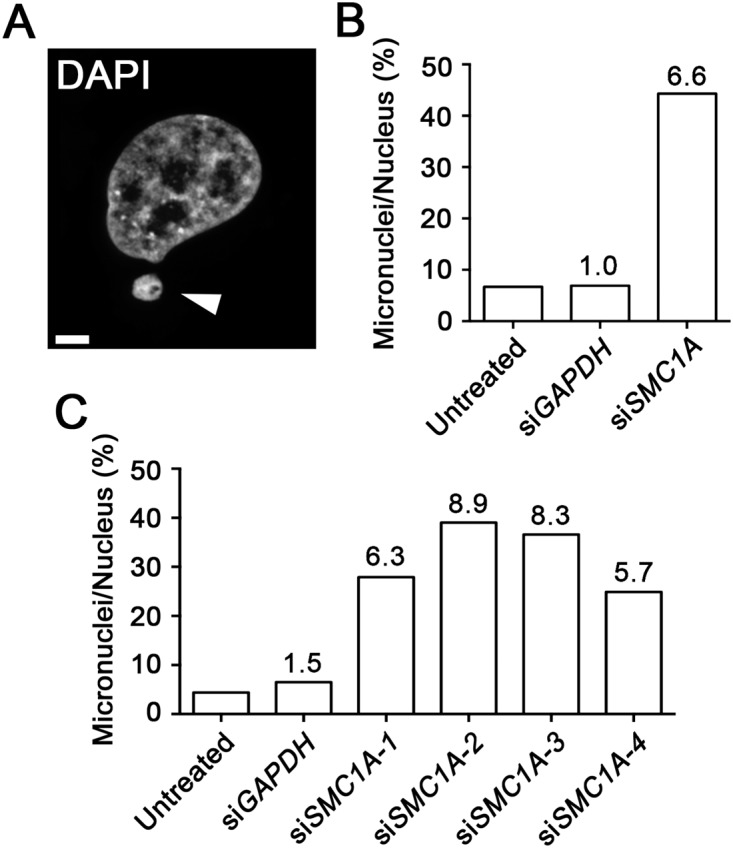
Micronucleus Formation is Induced Following *SMC1A* Silencing. (A) A representative high-resolution, 3D image highlighting a MN (arrowhead). Scale bar represents 5 μm. (B) Bar graph presenting the average number of micronuclei in each condition (x-axis), expressed as a percentage of the total number nuclei analyzed. The fold-increase relative to the untreated control is indicated above each column. (C) Bar graph depicting the average number of micronuclei following *SMC1A* silencing with individual siRNA duplexes and controls (expressed as a percent). The fold-increase relative to the untreated control is indicated above each column.

We next sought to develop an image-based approach to evaluate the appearance of micronuclei, a classical hallmark of CIN (Fig 3A). To do so, we investigated whether increases in MN formation accompanied *SMC1A* silencing. J21 cells were transiently transfected as above with siRNA duplexes targeting either *SMC1A* or *GAPDH*, or left untreated. Following a four-day incubation period, cells were fixed, counterstained, and imaged as above. The number of micronuclei in each condition was scored, and is expressed as a percentage of the total number of nuclei analyzed (Fig 3B). Overall, there was a 6.6-fold increase in the number of micronuclei observed within the *SMC1A*-silenced population (44.3%) relative to GAPDH-silenced (6.9%) or untreated (6.7%) controls. To ensure that the increase was not the result of off-target effects, micronuclei were enumerated following transfection with each of the four individual *SMC1A* siRNAs, as above. In agreement with the pooled approach, a marked increase in MN formation was observed following *SMC1A* silencing (24.9–39.0% micronuclei) compared to untreated (4.4%) or *GAPDH*-silenced (6.5%) cells (Fig 3C). As above, differences in MN formation occurred between the various *SMC1A* siRNAs that likely reflects the variability in silencing efficiency and/or the biological variation that is inherent within the CIN phenotype. Thus, these data validate the use of MN enumeration as a surrogate marker for CIN in J21 cells.

To verify that the increases in MN formation described above were not exclusive to the J21 cells, analogous experiments were performed in hTERT cells with similar, albeit less pronounced findings (S1 Fig). Overall, there was a 2.0-fold increase in MN formation following *SMC1A* silencing (5.4%) compared to untreated (2.7%) and *GAPDH*-silenced (3.0%) cells. Although beyond the scope of this study, the difference in MN formation observed between J21 and hTERT may reflect the different genetic contexts inherent within the transformed cancerous cell line and the immortalized diploid cell line, respectively. Collectively, the above data show that the increases in MN formation that accompany the silencing of *SMC1A* in both J21 and hTERT cells, and provides strong evidence supporting the use of MN formation as a surrogate marker of CIN.

## Discussion

In this study, we present a novel high-content, image-based approach capable of detecting three phenotypes commonly associated with CIN, namely changes in nuclear volume, changes in chromosome copy number (DsRED-LacI foci), and MN formation. SMC1A was purposefully selected as the positive control, as it has a critical role in chromosome cohesion that is now known to impact chromosome segregation, DNA replication and DNA double strand break repair[25–27]. Thus, diminished SMC1A expression was predicted to produce multiple, aberrant phenotypes that are readily detected using epi-fluorescence imaging microscopy. Using an siRNA-based approach, we identified statistically significant increases in nuclear volumes, alterations in chromosome 11 (11q13) copy number, and increases in MN formation following *SMC1A* silencing in J21 cells. Importantly, increases in nuclear volumes and MN formation were also observed in hTERT cells, highlighting the conserved nature of SMC1A function in chromosome stability in genetically diverse and different cellular contexts. As the DsRED-LacI foci enumeration assay is restricted to cells containing a *LacO* cassette (J21), these analyses were not performed in hTERT. However, it is conceivable that any cell line could be genetically modified to contain a *LacO* cassette, which would facilitate future investigations of a gene or genes in alternative cellular and genetic contexts. Collectively, the above data show that *SMC1A* silencing induces multiple CIN-related phenotypes that are detected through quantitative imaging microscopy. The results of this study validate the use of each individual assay to detect surrogate markers of CIN, and demonstrate the ability of this approach to screen for CIN phenotypes.

Intuitively, alterations in the physical space requirements associated with large increases in chromosome numbers (i.e. ploidy) will be reflected by corresponding changes in nuclear volume. The interrelationship of DNA content, nuclear size (i.e. area) and CIN has been known for decades[34–37, 45, 46], however the use of 3D nuclear volumes as a screen for CIN is entirely novel. In colorectal cancer for example, up to 85% of all tumors exhibit CIN[47], with most late-stage tumors harboring large increases in chromosome numbers, typically in the triploid to tetraploid range (i.e. 60–90 chromosomes)[48]. Due to the strong association of CIN and cancer, nuclear size can be employed to provide diagnostic and/or prognostic information. In lung cancer for example, increases in nuclear area correlate with tumor grade and stage[35, 45]. In support of the clinical associations highlighted above, our study demonstrates that significant increases in nuclear volumes accompany *SMC1A* silencing. The increases in nuclear volumes we observe in J21 and hTERT cells likely reflect the large-scale increases in chromosome numbers previously observed using a similar siRNA-based approach in HCT116 cells[18]. Thus, we identify *SMC1A* as a CIN gene in both J21 and hTERT cells, and conclude SMC1A is normally required to maintain chromosome stability in mammalian cells.

In general, we observe increases in nuclear sizes following *SMC1A* silencing rather than decreases, which is contrary to the segregation model of CIN (Fig 1A) that suggests both increases and decreases should be apparent. Although unknown, the underlying reason(s) are likely to be biological in nature rather than technical. One possibility is that increases in chromosome numbers (and the hundreds to thousands of genes they contain) are better tolerated than chromosome losses, which will result in haploinsufficiencies (single chromosome loss) and homozygous losses (both chromosomes lost) of key genes. By definition, the loss of an essential gene will result in death and the removal of these cells from the population under study. Indeed, further scrutiny of the original images identified a small subset of cells exhibiting apoptotic hallmarks including chromatin compaction and nuclear blebbing (data not shown). A second possibility is that *SMC1A* silencing affects additional pathways that underlie increases in nuclear volumes, such as DNA replication or double strand break repair (see [49]). While replication errors may amplify specific chromosomes and/or large chromosomal regions resulting in larger nuclei, DNA repair defects may produce acentric chromosome fragments that are not accurately segregated. Presumably, if these acentric fragments are incorporated within a daughter nucleus it will result in a larger DNA complement. Alternatively, if not incorporated within a daughter nucleus, micronuclei will be formed. Indeed, our MN analyses confirm that increases occurred following *SMC1A* silencing in both J21 and hTERT cells. However, it should be noted that MN formation occurred more readily within J21 cells than hTERT (6-fold versus 2-fold, respectively), which likely reflects the genetic differences between these cell types. While J21 cells are a sub-clone of a cancer cell line (HT1080) with known defects in DNA repair genes (e.g. *ERCC5*, *FANCC*, *MSH3*, and *WRN*[50]), hTERTs are an immortalized, non-cancerous cell line that does not contain any known defects. Therefore, the J21 cells likely exhibit diminished repair capabilities relative to the hTERT cells that render them hypersensitive to *SMC1A* silencing, and thus produce elevated numbers of micronuclei.

The development of a quantitative, image-based approach for the detection of CIN phenotypes is an important advancement over traditional approaches such as flow cytometry. The current approach facilitates rapid, visual assessment of the cells under investigation, which allows for simultaneous assessment of multiple CIN phenotypes. Importantly, visual examination of the cells from each condition can provide further information regarding the aberrant mechanisms that cause CIN (e.g. multipolar spindle formation, lagging chromosomes, multi-nucleated cells, *etc*.) that is not possible with flow cytometry. Further, this approach offers significant time, cost and labor savings over conventional cytogenetic approaches (e.g. mitotic chromosome spreads, spectral karyotyping), particularly since it is scalable to 96- and 384-well assay formats. Importantly, the current approach is performed on asynchronous cell populations, and in the absence of mitotic poisons including colcemid or nocodazole. Although these drugs are routinely employed by cytogeneticists to artificially increase mitotic indices, several studies have shown they can induce aneuploidy and MN formation[51, 52]. In addition, because CIN phenotypes are evaluated within interphase cells, our analyses are not restricted to a minor fraction of the entire population, and thus larger sample sizes (hundreds to thousands of nuclei) are obtained. We can therefore quantify CIN phenotypes that specifically arise during interphase such as increases in nuclear volumes associated with replication errors (e.g. endoreduplication). In any case, it is not difficult to envision how similar approaches could be directed towards the mitotic cells contained within the images to quantitatively assess additional CIN phenotypes, including lagging chromosomes and multi-polar spindles. Although this approach represents a significant advancement, we recognize that it does not replace the need and capabilities inherent within many classical cytogenetic approaches. Rather, we suggest that the multiplexed approach is highly amenable to screening siRNA/shRNA or chemical libraries to identify putative CIN candidates that will require subsequent validation using traditional cytogenetic approaches.

An image-based assay that can simultaneously assess multiple phenotypes associated with CIN is important, particularly in the context of a screen. Multiplexing of the nuclear volume assay with the DsRED-LacI and MN enumeration assays will help ensure maximal numbers of putative CIN genes are identified. As shown by Yuen *et al*[15] in budding yeast, the use of complementary assays is critical, particularly since some genes display assay specificity while others are detected by multiple assays. Furthermore, knowing whether or not newly identified CIN genes exhibit assay specificity will not only assist in prioritizing those candidates for subsequent study, but will also provide fundamental insight into the aberrant pathways implicated in CIN. This information will become particularly relevant as novel candidate CIN genes are identified, and their potential roles in the pathogenesis of human diseases such as cancer are explored. Thus, the identification of novel human CIN genes enabled with the current approach will provide critical insights into CIN and the aberrant biological mechanisms associated with highly aggressive, drug resistant, CIN-positive tumors. Ultimately, these insights may direct the future development of novel therapeutic strategies. Additionally, this new screening approach may hold prognostic or diagnostic value however its use in a clinical setting remains to be evaluated.

## Supporting Information

S1 Fig *SMC1A* Silencing in hTERT Induces Increases in CIN Phenotypes. (A) Western blot depicting SMC1A expression levels following silencing (si*SMC1A*-pool), with α-Tubulin as a loading control. (B) Box-and-whisker plot displaying the minimum, 25^th^ percentile, median, 75^th^ percentile and maximum nuclear volume values for each condition indicated on the x-axis. (C) Bar graph presents mean nuclear volumes (± SD). Student’s *t* tests were performed between the untreated hTERT cells and each of the conditions (si*GAPDH* and si*SMC1A*-pool). Statistically significant differences are identified by ****, (*p* <0.0001), and ns, (not significant). (D) Bar graph displays the average number of micronuclei as a percentage of the total number nuclei analyzed for each condition. Fold increases in MN formation for the *GAPDH* and *SMC1A*-silenced cells (si*SMC1A*-pool) relative to the untreated condition are displayed above each column.
(TIF)

S2 Fig Mitotic Chromosome Spreads Confirm *LacO* Array Locus and Copy Number. Representative mitotic chromosome spreads confirming the location (11q13) and copy number (2) of the *LacO* cassettes as visualized by DsRED-LacI binding. The arrowheads (yellow) identify the DsRED-LacI foci (red) within chromosome 11. Karyotypic analyses were conducted and the insert presented in the left panel provides a higher magnification of both copies of chromosome 11 with DsRED-LacI foci. Note that due to the normal loss of sister chromatid cohesion during mitosis, one DsRED-LacI focus is associated with each sister chromatid, which are not spatially resolved within interphase nuclei (G1, S-phase or G2).
(TIF)

S1 Table *SMC1A* Silencing Increases Mean Nuclear Volume in J21 Cells. (PDF)

S2 Table *SMC1A* Silencing by each siRNA Increases Mean Nuclear Volume in J21 Cells. (PDF)

S3 Table *SMC1A* Silencing Increases Mean Nuclear Volume in hTERT Cells. (PDF)

S4 Table *SMC1A* Silencing Induces Chromosome Content Changes in hTERT Cells. (PDF)

S5 Table The *LacO* Cassettes are Karyotypically Stable within J21 Cells. (PDF)
