# Supplementary figures and images for: A Novel Multiplexed, Image-Based Approach to Detect Phenotypes That Underlie Chromosome Instability in Human Cells
Source: PLoS One. 2015 Apr 20;10(4):e0123200. doi: 10.1371/journal.pone.0123200 (PMC4404342; doi:10.1371/journal.pone.0123200)

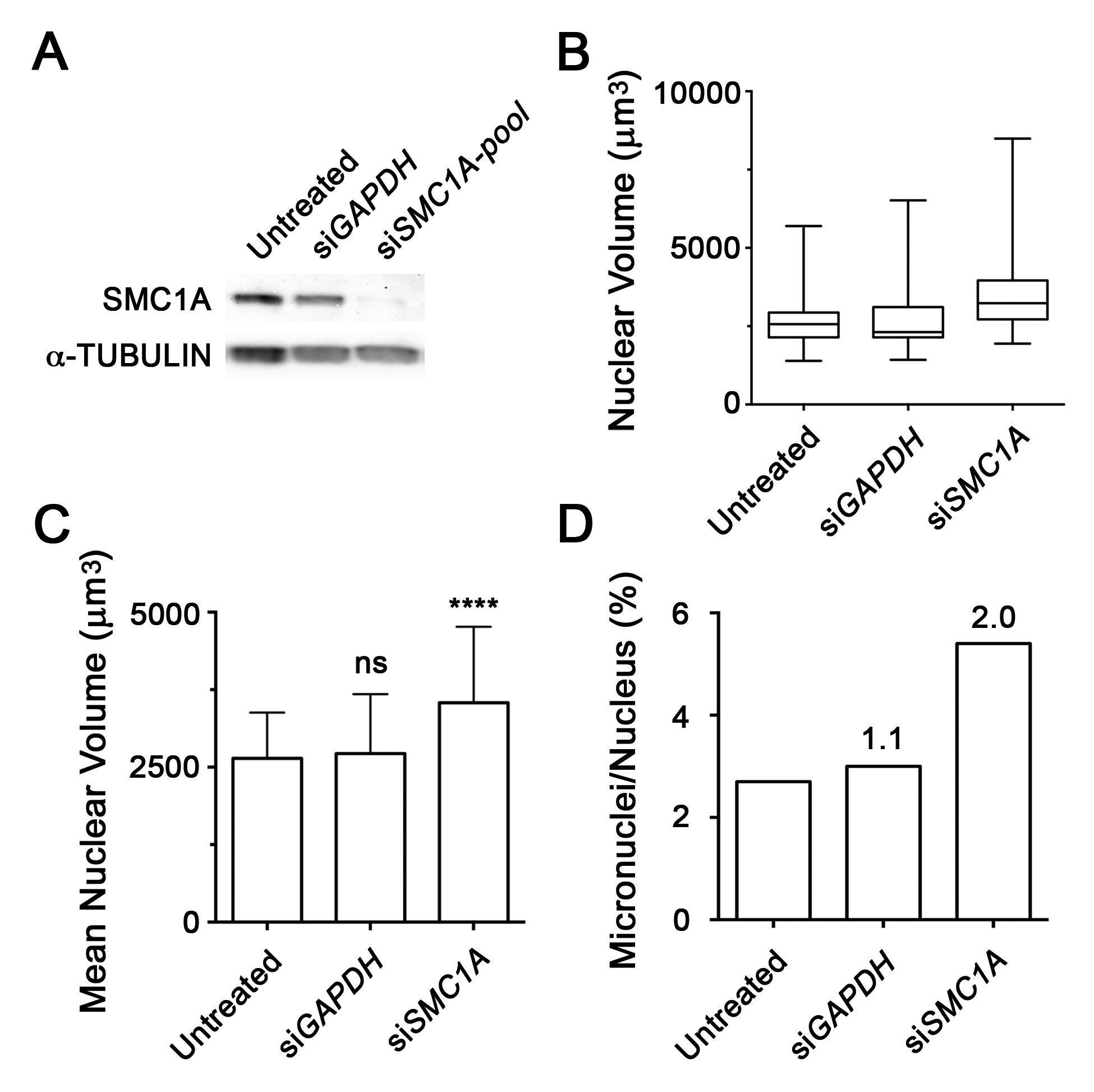

Supplement: S1 Fig — (A) Western blot depicting SMC1A expression levels following silencing (siSMC1A-pool), with α-Tubulin as a loading control. (B) Box-and-whisker plot displaying the minimum, 25th percentile, median, 75th percentile and maximum nuclear volume values for each condition indicated on the x-axis. (C) Bar graph presents mean nuclear volumes (± SD). Student’s t tests were performed between the untreated hTERT cells and each of the conditions (siGAPDH and siSMC1A-pool). Statistically significant differences are identified by ****, (p <0.0001), and ns, (not significant). (D) Bar graph displays the average number of micronuclei as a percentage of the total number nuclei analyzed for each condition. Fold increases in MN formation for the GAPDH and SMC1A-silenced cells (siSMC1A-pool) relative to the untreated condition are displayed above each column. (TIF) [file pone.0123200.s001.tif]

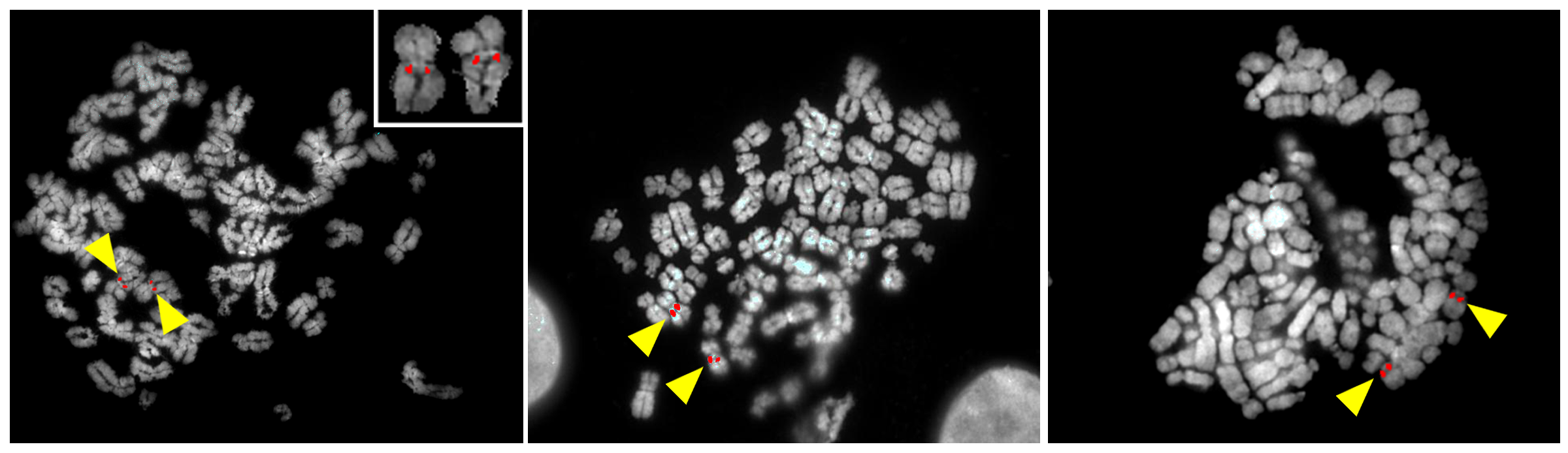

Supplement: S2 Fig — Representative mitotic chromosome spreads confirming the location (11q13) and copy number (2) of the LacO cassettes as visualized by DsRED-LacI binding. The arrowheads (yellow) identify the DsRED-LacI foci (red) within chromosome 11. Karyotypic analyses were conducted and the insert presented in the left panel provides a higher magnification of both copies of chromosome 11 with DsRED-LacI foci. Note that due to the normal loss of sister chromatid cohesion during mitosis, one DsRED-LacI focus is associated with each sister chromatid, which are not spatially resolved within interphase nuclei (G1, S-phase or G2). (TIF) [file pone.0123200.s002.tif]
