# Supplementary material for: A Novel Multiplexed, Image-Based Approach to Detect Phenotypes That Underlie Chromosome Instability in Human Cells
Source: PLoS One. 2015 Apr 20;10(4):e0123200. doi: 10.1371/journal.pone.0123200 (PMC4404342; doi:10.1371/journal.pone.0123200)
Supplement: S1 Table — (PDF) [file pone.0123200.s003.pdf]

**S1 Table. *SMC1A* Silencing Increases Mean Nuclear Volume in J21 Cells.**

| | # Nuclei | Mean Nuclear Volume ($\mu m^3$) | Standard Deviation | *p*-value[A] | *p*-value[B] | Fold Increase[C] |
|---|---|---|---|---|---|---|
| **Untreated** | 223 | 3008.1 | 592.6 | N/A[D] | 0.4602 | 1.0 |
| **si*GAPDH*** | 275 | 2955.9 | 907.7 | 0.4602 | N/A | 1.0 |
| **si*SMC1A*** | 273 | 4632.6 | 1608.7 | <0.0001 | <0.0001 | 1.5 |

[A]*p*-values obtained when comparing mean nuclear volumes to untreated control.
[B]*p*-values obtained when comparing mean nuclear volume to si*GAPDH* control.
[C]Fold increase values refer to the increase in mean nuclear volume relative to the untreated control.
[D]N/A; not applicable
