# Supplementary material for: A Novel Multiplexed, Image-Based Approach to Detect Phenotypes That Underlie Chromosome Instability in Human Cells
Source: PLoS One. 2015 Apr 20;10(4):e0123200. doi: 10.1371/journal.pone.0123200 (PMC4404342; doi:10.1371/journal.pone.0123200)
Supplement: S2 Table — (PDF) [file pone.0123200.s004.pdf]

S2 Table. ***SMC1A*** **Silencing by each siRNA Increases Mean Nuclear Volume in J21 Cells.**

	**# Nuclei**	**Mean Nuclear Volume (μm^3)**	**Standard Deviation**	***p*-value**	**Fold Increase[A]**
**Untreated**	207	3022.7	866.0	N/A[B]	1.0
**si*GAPDH***	191	3104.8	764.6	0.3184	1.0
**si*SMC1A-1***	247	3292.2	1063.4	0.0036	1.1
**si*SMC1A-2***	141	3412.9	1287.9	0.0008	1.1
**si*SMC1A-3***	194	3991.2	1474.4	<0.0001	1.3
**si*SMC1A-4***	128	4828.0	1477.5	<0.0001	1.6

[A]Fold increase values refer to the increase in mean nuclear volume relative to the untreated control.

[B]N/A; not applicable
