# Supplementary material for: A Novel Multiplexed, Image-Based Approach to Detect Phenotypes That Underlie Chromosome Instability in Human Cells
Source: PLoS One. 2015 Apr 20;10(4):e0123200. doi: 10.1371/journal.pone.0123200 (PMC4404342; doi:10.1371/journal.pone.0123200)
Supplement: S3 Table — (PDF) [file pone.0123200.s005.pdf]

S3 Table. ***S MC1A*** **Silencing Increases Mean Nuclear Volume in hTERT Cells.**

	# Nuclei	Mean Nuclear Volume (μm^3)	Standard Deviation	*p*-value[A]	*p*-value[B]	Fold Increase[C]
**Untreated**	157	2640.7	740.8	N/A[D]	0.4558	1.0
**si*GAPDH***	101	2720.1	959.1	0.4558	N/A	1.0
**si*SMC1A***	60	3539.5	1225.0	<0.0001	<0.0001	1.3

[A]*p*-values obtained when comparing mean nuclear volumes to untreated control.
[B]*p*-values obtained when comparing mean nuclear volumes to si*GAPDH* control.
[C]Fold increase values refer to the increase in mean nuclear volume relative to the untreated control.
[D]N/A; not applicable
