# Supplementary material for: A Novel Multiplexed, Image-Based Approach to Detect Phenotypes That Underlie Chromosome Instability in Human Cells
Source: PLoS One. 2015 Apr 20;10(4):e0123200. doi: 10.1371/journal.pone.0123200 (PMC4404342; doi:10.1371/journal.pone.0123200)
Supplement: S4 Table — (PDF) [file pone.0123200.s006.pdf]

S4 Table. *SMC1A* Silencing Induces Chromosome Content Changes in hTERT Cells.

Condition	# Spreads	Spreads with Abnormal Chromosome Count (%)	*p*-value[A]	Fold Increase[B]
**Untreated**	100	11	N/A[C]	N/A
**si*GAPDH***	100	9	>0.9999	0.82
**si*SMC1A***	100	42	0.0112	3.82

[A]A Kolmogorov-Smirnov test was used to calculate statistical significance between chromosome number distributions from each condition and the untreated (negative) control. A *p*-value <0.05 is considered significant.

[B]Fold increase refers to the increase in the percentage of spreads harboring an abnormal chromosome number (≠ 46) relative to the untreated control.

[C]N/A; not applicable
