# Supplementary material for: A Novel Multiplexed, Image-Based Approach to Detect Phenotypes That Underlie Chromosome Instability in Human Cells
Source: PLoS One. 2015 Apr 20;10(4):e0123200. doi: 10.1371/journal.pone.0123200 (PMC4404342; doi:10.1371/journal.pone.0123200)
Supplement: S5 Table — (PDF) [file pone.0123200.s007.pdf]

S5 Table. The *LacO* Cassettes are Karyotypically Stable within J21 Cells.

Time (weeks)	Number of Interphase Cells Evaluated	Percentage of Cells with Two DsRED-LacI Foci
0	103	79.6
6	175	78.3
